# Cetuximab ameliorates suppressive phenotypes of myeloid antigen presenting cells in head and neck cancer patients

**DOI:** 10.1186/s40425-015-0097-6

**Published:** 2015-11-17

**Authors:** Jing Li, Raghvendra M. Srivastava, Abhinav Ettyreddy, Robert L. Ferris

**Affiliations:** Department of Pharmacology and Pharmaceutical Sciences, School of Medicine, Tsinghua University, Beijing, China; Department of Otolaryngology, University of Pittsburgh, Pittsburgh, PA USA; School of Medicine, University of Pittsburgh, Pittsburgh, PA USA; Department of Immunology, University of Pittsburgh, Pittsburgh, PA USA; Cancer Immunology Program, University of Pittsburgh Cancer Institute, Pittsburgh, PA USA; Hillman Cancer Center Research Pavilion, 5117 Centre Avenue, Room 2.26b, Pittsburgh, PA 15213-1863 USA

**Keywords:** Monoclonal antibody, EGFR, Myeloid derived suppressor cells, Macrophages

## Abstract

**Background:**

Myeloid-derived suppressor cells (MDSC) and M2 monocytes/macrophages are two types of suppressive myeloid antigen presenting cells that have been shown to promote tumor progression and correlate with poor prognosis in cancer patients. Tumor antigen specific monoclonal antibodies (mAb) have emerged as important agents for cancer therapy. In addition to the direct inhibition of tumor growth, the Fc portions of the therapeutic mAbs, such as the IgG1 portion of the anti-epidermal growth factor receptor (EGFR) mAb cetuximab, might interact with the Fc-gamma receptors (FcγR) on myeloid cells and modulate their suppressive activity.

**Methods:**

Patients with locally advanced head and neck squamous cell carcinoma (HNSCC) on the UPCI 08–013 NCT01218048 trial were treated with single-agent cetuximab before surgery. Blood were collected pre- and post-cetuximab treatment to analyze frequency of monocytic MDSC (CD11b^+^CD14^+^HLA-DR^lo/-^), granulocytic MDSC (LIN^−^CD11b^+^CD15^+^) and CD11b^+^CD14^+^HLA-DR^hi^ monocytes by flow cytometry. Besides, CD11b^+^CD14^+^HLA-DR^hi^ monocytes were sorted for qPCR analysis of IL-10 and IL-12B transcripts. MDSC were generated in vitro with or without coated hIgG1 and tested for suppressive activity in mixed leukocyte reaction (MLR). Naïve monocytes from HNSCC patients co-cultured with tumor cell lines in the presence of cetuximab or hIgG1 were analyzed for M1/2 surface markers and cytokines.

**Results:**

We observed significantly increased monocytic MDSC in non-responders and decreased granulocytic MDSC in responders after cetuximab treatment. In addition, circulating CD11b^+^CD14^+^HLA-DR^hi^ monocytes of cetuximab responders displayed attenuated M2 polarization, with decreased CD163^+^ expression and IL-10 transcripts after cetuximab treatment. This beneficial effect appeared to be FcγR dependent, since CD16 ligation reproduced the reversal of suppressive activity of MDSC *in vitro*. CD14^+^ naïve monocytes from the co-cultures of tumor cells, cetuximab and HNSCC patient PBMC or purified monocytes were skewed to an M1-like phenotype, with increased expression of HLA-DR, CD86 and production of IL-12 p70. Likewise, reduced M2 features (expression of CD163 and production of IL-10) were found after crosslinking CD16 on the surface of monocytes to cetuximab-coated tumor cells.

**Conclusion:**

Our studies demonstrate a novel function of cetuximab in ameliorating suppressive phenotypes of FcγR bearing myeloid cells in cancer patients, which is associated with better clinical outcome of cetuximab-treated patients.

**Clinical trial registry:**

#NCT01218048. Registered 7 October 2010.

**Electronic supplementary material:**

The online version of this article (doi:10.1186/s40425-015-0097-6) contains supplementary material, which is available to authorized users.

## Background

Despite recent advances in surgery, chemotherapy and radiotherapy, the overall 5-year survival rate for head and neck squamous cell carcinoma (HNSCC) remains at about 50 %. Cetuximab, an anti-epidermal growth factor receptor (EGFR) monoclonal antibody, has become an important targeted therapy for multiple types of solid tumors and has been FDA approved for treating locally advanced and recurrent/metastatic HNSCC. However, response rates are only 15–20 % [1, 2], and its mechanism of action has not been determined [3, 4]. Besides inhibition of EGFR tyrosine phosphorylation and the subsequent downstream signaling, the human IgG1 Fc portion of cetuximab can activate NK cell-mediated antibody dependent cell-mediated cytotoxicity (ADCC) in a Fc-gamma receptor (FcγR)-dependent manner [5], triggering enhanced antigen presentation and resulting in an adaptive tumor-specific immune response [6]. However, since FcγRs are also widely expressed on myeloid antigen presenting cells (APC) in addition to NK cells, cetuximab is also likely to modulate their phenotype and function.

Myeloid APC with potent immunosuppressive activities, including myeloid-derived suppressor cells (MDSC) and M2 macrophages (MΦ)/monocytes (Mo), are induced by multiple tumor-derived soluble factors from the tumor microenvironment, and are utilized by the tumor cells to facilitate immune evasion and to promote tumor progression [7–9]. Increased abundance of MDSC and M2 MΦ/Mo is associated with poor clinical outcome [10–12] and is a major obstacle to existing cancer therapies [8, 13]. Therefore, they represent novel therapeutic targets for manipulating the host immune response against tumor cells. MDSC is a heterogeneous population of immature myeloid cells first identified in tumor-bearing mice as CD11b^+^Gr-1^+^ and can be subdivided into two major classes: granulocytic and monocytic MDSC. Granulocytic MDSC inhibit T cell responses mainly through reactive oxygen species (ROS) [14, 15], while monocytic MDSC suppresses T cell by depleting L-arginine via arginase-I and iNOS [16, 17]. In HNSCC patients, LIN^−^CD11b^+^CD15^+^ cells have been characterized as granulocytic MDSC [18], while CD11b^+^CD14^+^HLA-DR^lo/-^ cells have been identified as monocytic MDSC with immunosuppressive functions [19]. M1/M2 polarization plays an important role in modulating anti-tumor immune responses. M1, the classically activated MΦ/Mo induced by IFN-γ and bacterial products, display high antigen presentation efficiency due to upregulation of MHCII (HLA-DR) and co-stimulatory molecules (CD80 and CD86), express high levels of IL-12 and low IL-10, and thus activate Tc1/Th1 anti-tumor responses. In contrast, M2, the alternatively activated MΦ/Mo induced by anti-inflammatory cytokines, produce high levels of IL-10 and low levels of IL-12, and thus inhibit development of Th1 cells and CTL responses and facilitate tumor progression [17]. Therefore, polarization of MΦ/Mo towards an M1 phenotype is indispensable to enhance efficacy of anti-tumor immunotherapy.

Since CD16 receptor binds to the Fc portion of cetuximab and its intracellular immunoreceptor tyrosine-based activation motif (ITAM) has been suggested to induce DC maturation [20], we hypothesized that cetuximab can skew these myeloid cells away from immunosuppressive capacity of MDSC and M2 cells. In this study, we investigated the effects of cetuximab on the well-reported abundance of granulocytic and monocytic MDSC as well as the M1/M2 polarization of monocytes in the peripheral circulation in HNSCC patients, taking advantage of a phase II trial of neoadjuvant cetuximab. In addition, we also utilized *in vitro* generated MDSC in the presence or absence of CD16 ligation in a suppression assay and co-culture of tumor cells and PBMC or purified monocytes from HNSCC patients with or without cetuximab, to further investigate the mechanism of cetuximab mediated MDSC activity.

## Results

### Circulating monocytic MDSC increase in cetuximab non-responding patients

**Fig. 1:**
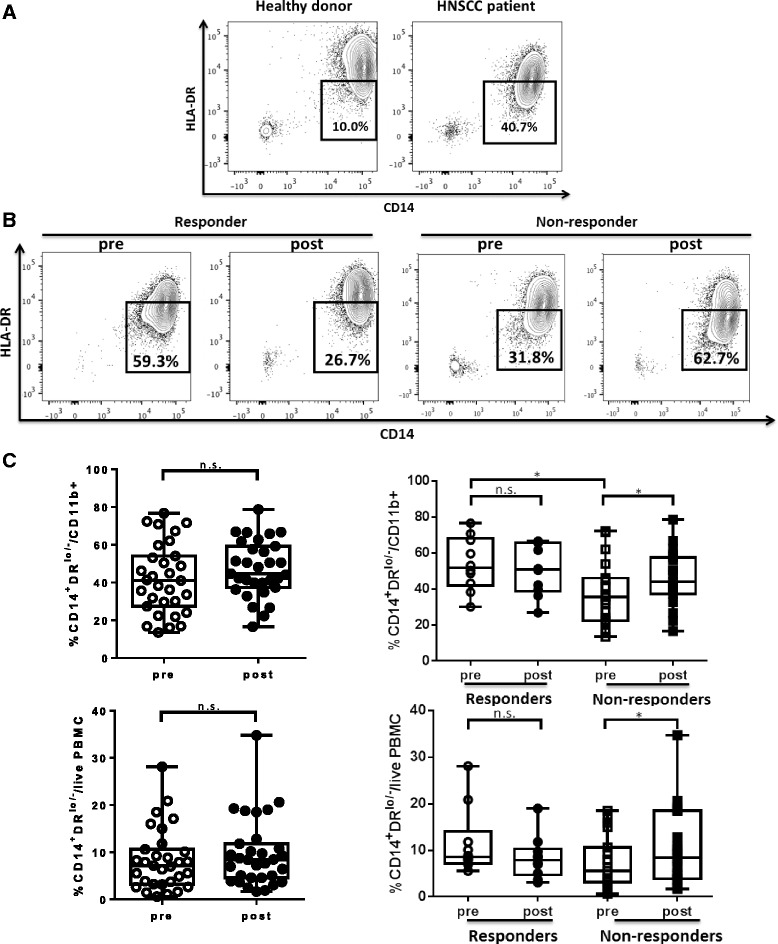
Circulating monocytic MDSC (CD11b^+^CD14^+^HLA-DR^lo/-^) increased after cetuximab treatment in non-responders after cetuximab neoadjuvant therapy. Levels of monocytic MDSC (CD11b^+^CD14^+^HLA-DR^lo/-^) in the peripheral blood of healthy donors versus HNSCC patients and HNSCC patients pre- and post-cetuximab treatment were analyzed by flow cytometry. **a** Representative figures showing frequency of CD14^+^HLA-DR^lo/-^ cells in CD11b^+^ mononuclear cells in peripheral blood from healthy donors and HNSCC patients. The mean LIN^−^CD11b^+^ cells from healthy donors and HNSCC patients were not statistically different. **b** Representative figures showing percentage of CD14^+^HLA-DR^lo/-^ cells in circulating CD11b^+^ cells from responders and non-responders of UPCI 08–013 trial before and after cetuximab treatment. **c** Summary data of frequency of CD14^+^HLA-DR^lo/-^ cells in CD11b^+^ PBMC or in total live PBMC pre- and post-cetuximab treatment in the total 40 HNSCC patients (left panel) and in responders (*n* = 10) and non-responders (*n* = 19) of UPCI 08–013 trial (right panel). Statistical significance was determined by Wilcoxon matched-pairs signed rank tests for the same patients pre- and post-cetuximab treatment and Mann Whitney test for baseline levels in responders versus non-responders. *p < 0.05

Since monocytic myeloid-derived suppressor cells (MDSC) have been shown to be enriched in the peripheral blood of cancer patients, we investigated the population of circulating monocytic MDSC, the other subset of MDSC enriched in HNSCC patients, characterized as CD14^+^HLA-DR^lo/-^, in HNSCC patients on the UPCI 08–013 trial, a cetuximab single agent trial in which the patients received weekly doses of cetuximab for 3 to 4 weeks before surgery [19]. First, we examined the baseline frequency of circulating CD14^+^HLA-DR^lo/-^ in the CD11b^+^ compartment in the cohort of patients on the 08–013 trial of neoadjuvant cetuximab, as compared with healthy donors by flow cytometry (gating strategy shown in Additional file 1: Figure S1A). As expected, stage III/IV HNSCC patients showed significantly higher CD14^+^HLA-DR^lo/-^ cells in circulating CD11b^+^ cells at baseline compared with healthy donors (Fig. 1a). We then tested whether cetuximab treatment altered the level of circulating monocytic MDSC in the HNSCC patients.

Interestingly, a significant increase of monocytic MDSC in CD11b^+^ cells (*p* = 0.01) and in whole peripheral blood mononuclear cells (PBMC) (*p* = 0.01) was observed in non-responder patients after cetuximab treatment. Surprisingly, the baseline level of CD14^+^HLA-DR^lo/-^ cells within CD11b^+^ PBMC was higher in responders than in non-responders (*p* = 0.02). However, the cetuximab clinical responders did not show upregulation of circulating monocytic MDSC. On the contrary, 7 of the 10 responders had decreased levels of monoctyic MDSC in the peripheral circulation post-cetuximab, but this finding did not reach statistical significance (Fig. 1b and c). The baseline levels of CD16 expression on circulating monocytic MDSC are similar between responders and non-responders (Additional file 1: Figure S2), indicating different clinical responses to cetuximab treatment are not due to different baseline level of CD16. Our data indicates that cetuximab can overcome the enrichment of circulating monocytic MDSC in patients with advanced HNSCC, with the possibility of decreasing these cells in a subset of clinical responders.

### Decreased circulating granulocytic MDSC in HNSCC patients after cetuximab treatment

**Fig. 2:**
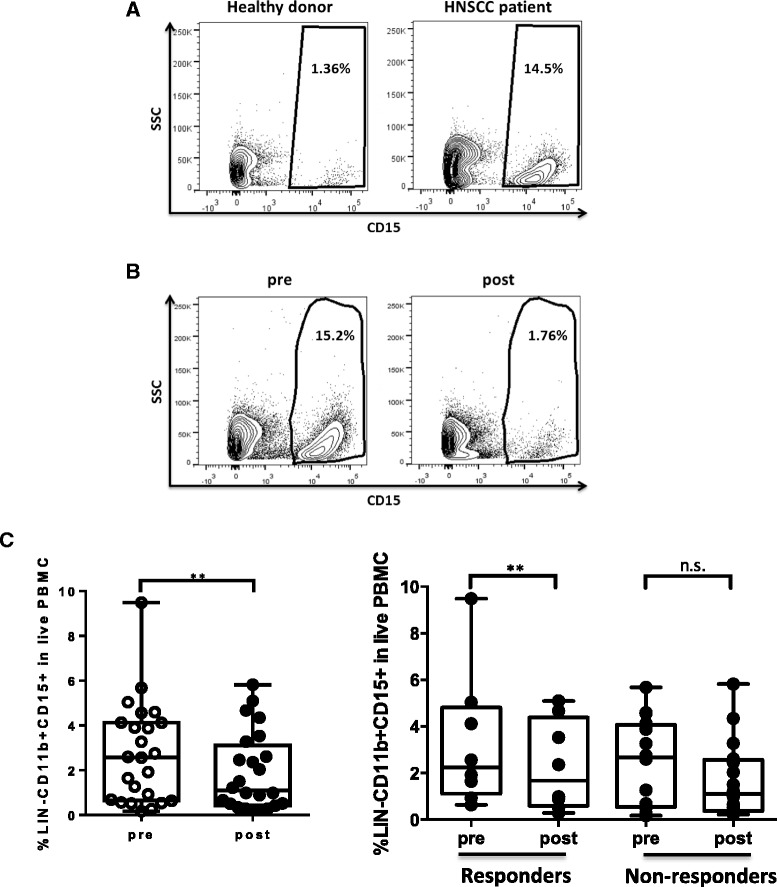
Granulocytic MDSC (LIN^−^CD11b^+^CD15^+^) in the peripheral blood of HNSCC patients decreased after cetuximab treatment. Levels of granulocytic MDSC (LIN^−^CD11b^+^CD15^+^) in the peripheral blood of healthy donors versus HNSCC patients and patients pre- and post-cetuximab treatment were analyzed by flow cytometry. **a** Representative figures showing frequency of CD15^+^ cells in LIN^−^CD11b^+^ mononuclear cells in peripheral blood from healthy donors and HNSCC patients. **b** Representative figures showing percentage of CD15^+^ cells in circulating LIN^−^CD11b^+^ cells in HNSCC patients before and after cetuximab treatment. **c** Summary data of frequency of LIN^−^CD11b^+^CD15^+^ cells in live PBMC pre- and post-cetuximab treatment in the total 24 HNSCC patients (left panel, **p < 0.01) and in responders (*n* = 8, **p < 0.01) and non-responders (*n* = 16) of UPCI 08–013 trial (right panel). Statistical significance was determined by Wilcoxon matched-pairs signed rank tests. **p < 0.01. *p* > 0.05 was considered to be not significant (n.s.)

Having demonstrated the changes of monocytic MDSC in the patients of the 08–013 trial, we next studied the abundance of circulating granulocytic MDSC, the other subset of MDSC, in our cohort of HNSCC patients. First, we compared the frequency of granulocytic MDSC (LIN^−^CD11b^+^CD15^+^) in the circulation of healthy donors and HNSCC patients prior to cetuximab treatment (gating strategies shown in Additional file 1: Figure S1A). Consistent with previous report [18], we observed much higher levels of circulating LIN^−^CD11b^+^CD15^+^ in HNSCC patients than in healthy donors (Fig. 2a, *p* < 0.01). Next, we examined the frequency of circulating granulocytic MDSC in this cohort of HNSCC patients pre- and post-cetuximab treatment. Interestingly, the overall percentage of granulocytic MDSC significantly decreased in the circulation of the 24 patients tested (*p* = 0.008). However, a significant and consistent decrease in the abundance of circulating granulocytic MDSC was observed only in the cetuximab responders (*n* = 8, *p* = 0.008) as compared to cetuximab-resistant patients (*n* = 16) (Fig. 2b and c). Our findings suggest that cetuximab treatment might modulate the development of granulocytic MDSC in HNSCC patients.

### Decreased CD163^+^ cells and IL-10 transcripts in circulating CD11b^+^CD14^+^HLA-DR^hi^ monocytes in cetuximab-treated responders

**Fig. 3:**
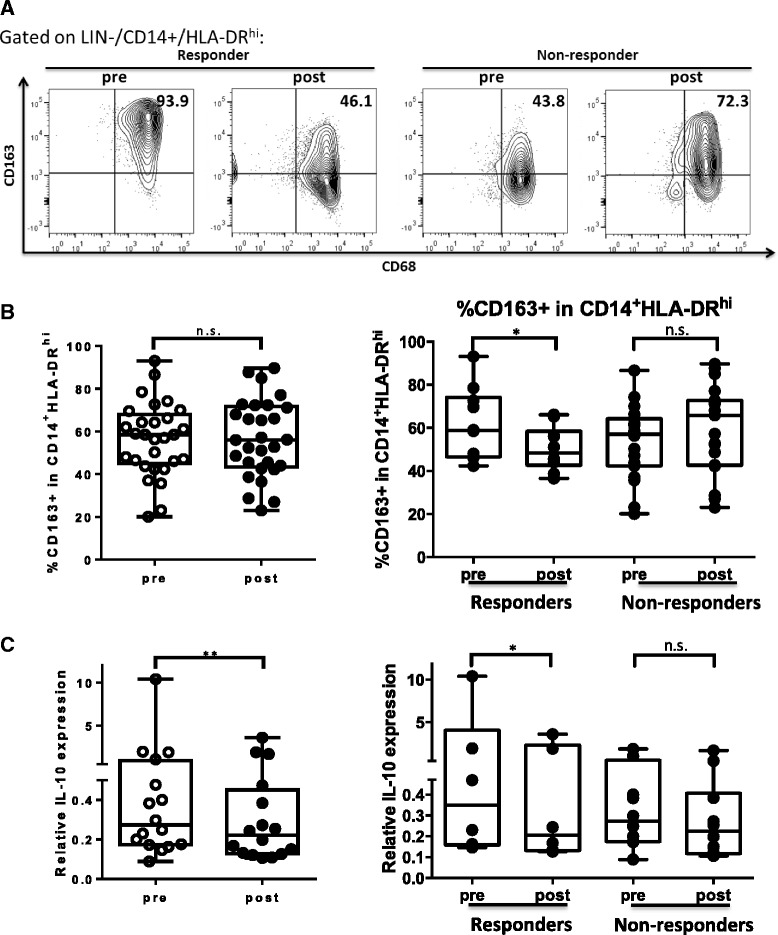
Cetuximab responders showed decreased CD163^+^ cells and IL-10 transcripts in circulating CD11b^+^CD14^+^HLA-DR^hi^ monocytes after cetuximab treatment. **a** Representative figures showing percentage of CD68^+^CD163^+^ cells in circulating CD11b^+^CD14^+^HLA-DR^hi^ cells from responders and non-responders during, pre- and post-neoadjuvant cetuximab treatment. **b** Summary data of frequency of CD163^+^ cells in CD11b^+^ CD14^+^HLA-DR^hi^ monocytes in the peripheral circulation pre- and post-cetuximab treatment in the total 29 HNSCC patients (left panel) and in responders (*n* = 10, *p < 0.05) and non-responders (*n* = 19) of UPCI 08–013 trial. Circulating CD11b^+^CD14^+^HLA-DR^hi^ monocytes were sorted from PBMC of 14 cetuximab treated patients and RNA was isolated for real time quantitative PCR analysis of IL-10 transcripts. **c** Summary data showing relative IL-10 expression in circulating CD11b^+^CD14^+^HLA-DR^hi^ monocytes pre- and post-cetuximab treatment in the total 14 HNSCC patients tested (left panel, *p < 0.05) and in responders (*n* = 6, *p < 0.05) and non-responders (*n* = 8) in the neoadjuvant cetuximab trial (right panel). The quantity of each cDNA sample was normalized by GUSB. All of the experiments were performed in triplicate. Statistical significance was determined by Wilcoxon matched-pair signed rank tests. *p < 0.05. *p* > 0.05 was considered to be not significant (n.s.)

Next, we investigated another major subpopulation of CD14^+^ circulating monocytes, specifically CD14^+^HLA-DR^hi^ cells that are likely to be precursors of tissue macrophages, to analyze whether cetuximab treatment alters their M1/M2 phenotypic skewing in HNSCC patients on the cetuximab neoadjuvant trial. First, we analyzed expression of CD163, a scavenger receptor usually used as an M2 surface marker and up-regulated by anti-inflammatory cytokines such as IL-10 and IL-6 [21], on circulating CD11b^+^CD14^+^HLA-DR^hi^ monocytes. Although there was no significant difference in frequency of CD163^+^ cells in CD11b^+^CD14^+^HLA-DR^hi^ monocytes in the circulation of HNSCC patients tested overall (*n* = 29) pre- and post-cetuximab treatment, we observed a significant decrease in percentage of CD163^+^ cells in circulating CD11b^+^CD14^+^HLA-DR^hi^ monocytes in the cetuximab clinical responders (*n* = 9, *p* = 0.049) (Fig. 3a and b). These findings suggest that cetuximab treatment might lessen M2 skewing of circulating monocytes or potentially induce a more beneficial anti-inflammatory environment in the HNSCC patients’ tumors.

In order to investigate the M1/M2 skewing of circulating CD11b^+^CD14^+^HLA-DR^hi^ monocytes in the cetuximab-treated patients more definitively, we sorted the CD11b^+^CD14^+^HLA-DR^hi^ from the patient PBMC pre- and post-cetuximab treatment by FACS sorting and purified the RNA for real time qPCR analysis of IL-12B (produced by M1 or mature dendritic cells) and IL-10 (mainly produced by M2) transcripts. While IL-12 transcripts in circulating CD11b^+^CD14^+^HLA-DR^hi^ monocytes were too low to be determined, expression of IL-10 transcripts was down-regulated in CD11b^+^CD14^+^HLA-DR^hi^ monocytes from HNSCC patient PBMC (*n* = 14, *p* = 0.02) after cetuximab treatment. Moreover, circulating CD11b^+^CD14^+^HLA-DR^hi^ monocytes from cetuximab responders showed consistent and significant decreased IL-10 transcripts post-cetuximab treatment (*n* = 6, *p* = 0.03), while some of the cetuximab non-responders (*n* = 8) manifested increased IL-10 transcripts in circulating CD11b^+^CD14^+^HLA-DR^hi^ monocytes (Fig. 3c). These findings indicate that the CD11b^+^CD14^+^HLA-DR^hi^ monocytes in the peripheral circulation of cetuximab-treated patients displayed less M2 skewing, including with decreased expression of IL-10 transcripts.

### FcγR ligation can reverse the suppressive effects of IL-6 + GM-CSF-induced MDSC on T cell proliferation

**Fig. 4:**
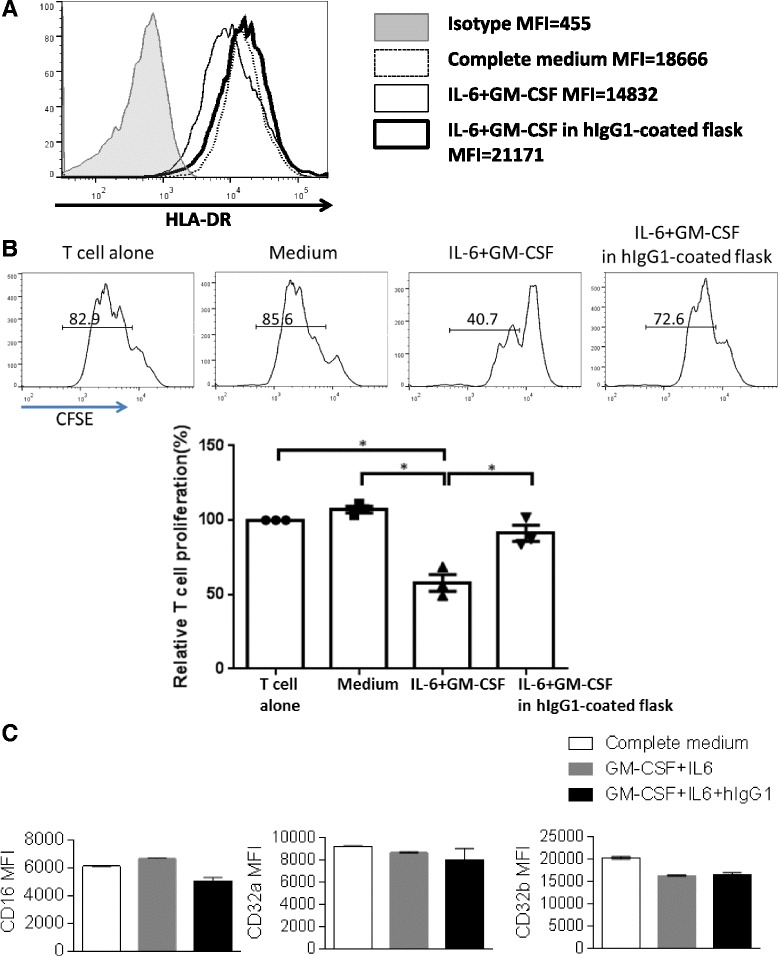
Ligation to flask-coated hIgG1 could reverse the suppressive effects of IL-6 + GM-CSF-induced MDSC on T cell proliferation. Total PBMC were isolated from peripheral blood of 3 healthy donors, plated in regular flasks or hIgG1-coated flasks (5 × 10^5^/mL) supplied with or without 10 ng/mL IL-6 and 10 ng/mL GM-CSF and cultured for 7 days. Then cells were harvested and surface markers were analyzed by flow cytometry. **a** Representative figure showing expression levels of HLA-DR on CD33^+^ myeloid cells from the indicated conditions. CD33^+^ cells were also isolated by FACS sorting and co-cultured with CFSE-labeled autologous T cells (10^5^ cells/well) at a 1:2 ratio. T cell stimulation was provided by anti-CD3/CD28 magnetic beads (bead: cell = 1:1). CFSE dilution was analyzed by flow cytometry for T cell proliferation after 3 days. **b** Summary data of proliferation of T cells co-cultured with CD33^+^ cells from the above cultures is shown. Proliferation of T cells in each condition was normalized to the matched T cell alone condition (100 %). The graph presents the mean ± SEM from 3 different healthy donors. Statistical significance was determined by one-way ANOVA followed by Tukey multiple comparison test. *p < 0.05. **c** Summary data showing expression level of surface CD16, CD32a and CD32b on CD33^+^ myeloid cells from the indicated conditions

Since FcγR are widely expressed on myeloid cells, and the Fc portion of cetuximab, human IgG1 (hIgG1), can bind to FcγR such as FcγRIII (CD16) and trigger the downstream signaling, we hypothesized that cetuximab may directly act on the FcγR on the myeloid cells to ameliorate their suppressive phenotypes in cancer patients. In order to test this possibility, we took advantage of an established *in vitro* induction procedure for MDSC from peripheral blood of healthy donors to test whether ligating the FcγR to hIgG1 Fc domains changes the phenotypes and suppressive functions of the *in vitro* induced MDSC. We coated hIgG1 (10 μg/mL) to polystyrene flasks and plated the PBMC isolated from healthy donors (*n* = 3) in the hIgG1-coated flasks or uncoated flasks, in the presence or absence of 10 ng/mL IL-6 and GM-CSF for 7 days. Surface markers were analyzed by flow cytometry and CD33^+^ myeloid cells were isolated and cultured with the CFSE-labeled autologous T cells to test suppressive activity. Expression of HLA-DR was downregulated in the presence of IL-6 and GM-CSF, which is consistent with the expectation that MDSC have deficient HLA-DR expression, while ligation of FcγR by polyvalent hIgG1 increased HLA-DR expression in these MDSC inducing conditions (Fig. 4a). Furthermore, while IL-6 and GM-CSF-induced MDSC strongly suppressed proliferation of autologous T cells (Fig. 4b, *p* = 0.03), the presence of hIgG1 coated surfaces during the *in vitro* induction ameliorated the suppressive effects of cytokine-induced MDSC on T cells (Fig. 4b, *p* = 0.02). Notably, we observed a concomitant down-regulation of CD16, but not CD32a or CD32b on the surface of CD33^+^ cells cultured in the hIgG1-coated flask (Fig. 4c), indicating hIgG1 mainly interacts with CD16 but not CD32a/b on the myeloid cells. Taken together, our data indicates that cetuximab can skew circulating myeloid cells away from MDSC-like suppressive phenotypes toward an FcγR-dependent manner.

### Cetuximab skews monocytes co-cultured with tumor cells to an M1 instead of M2 phenotype

**Fig. 5:**
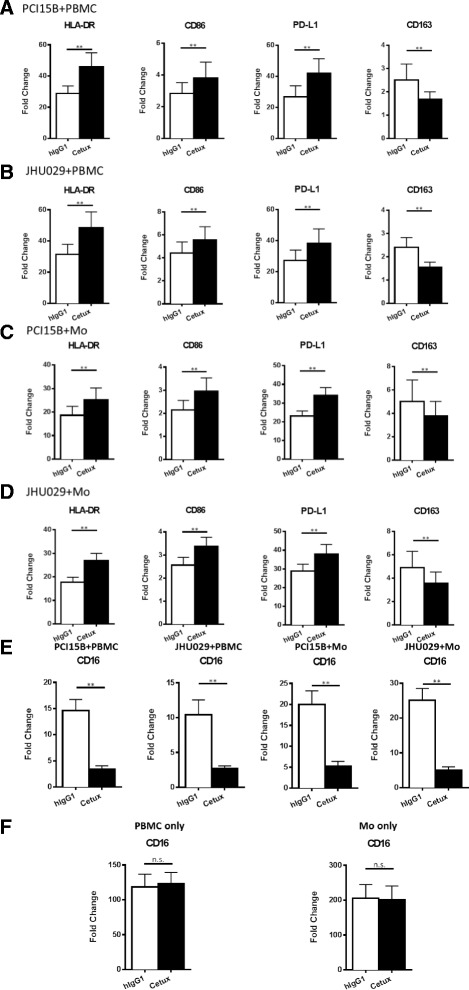
Expression of HLA-DR, CD86 and PD-L1 were increased, while expression of CD163 was decreased on CD14^+^ monocytes,concomitant with downregulation of CD16, in the co-cultures of tumor cells and PBMC or purified monocytes in the presence of cetuximab. Total PBMC or purified monocytes from HNSCC patients with new active diseases (*n* = 9) were co-cultured with PCI15B or JHU029 for 3 days with 10 μg/mL hIgG1 or cetuximab before analysis of surface markers on CD14^+^ monocytes by flow cytometry. Summary graphs showing fold changes (MFI normalized to each isotype) of HLA-DR, CD86, PD-L1 and CD163 on CD14^+^ monocytes from PCI15B+ PBMC (**a**), JHU029+ PBMC (**b**), PCI15B+ purified monocytes (**c**) and JHU029+ purified monocytes (**d**) co-cultures supplied with 10 μg/mL hIgG1 or cetuximab. Surface expression of CD16 of gated CD14^+^ monocytes from the co-cultures of tumor cells and PBMC/monocytes indicated above or cultures of PBMC/monocytes alone without tumor cells was analyzed by flow cytometry. Summary graphs of fold change of CD16 on CD14^+^ monocytes from the co-cultures of tumor cells and PBMC/monocytes **(e**) and cultures of PBMC/monocytes in the absence of EGFR^+^ tumor cells (**f**) are shown. The graphs present the mean ± SEM from advanced disease HNSCC patients (*n* = 9). Statistical significance was determined by Wilcoxon matched-pair signed rank tests. **p < 0.01

Having demonstrated that CD11b^+^CD14^+^HLA-DR^hi^ monocytes in the peripheral circulation of cetuximab responders showed attenuated M2 skewing with decreased CD163^+^ cells and IL-10 transcripts after cetuximab treatment, we next investigated whether cetuximab alters M1/M2 skewing of naïve monocytes *in vitro*. Total PBMC or purified monocytes from HNSCC patients with new active diseases (*n* = 9) were co-cultured with PCI15B or JHU029 (HNSCC cell lines with high EGFR expression) for 3 days with 10 μg/mL cetuximab or hIgG1 (isotype control), before analysis of surface markers on CD14^+^ monocytes by flow cytometry. As expected, expression of HLA-DR and CD86, which are well known as markers of M1 macrophages, were up-regulated on CD14^+^ monocytes within PBMC co-cultured with tumor cells in the presence of cetuximab (*p* = 0.004). In addition, upregulation of PD-L1, which correlates with the activation status of monocytes [22], was more pronounced on monocytes incubated with cetuximab versus control mAb (*p* = 0.004). Moreover, naïve monocytes co-cultured with tumor cells showed decreased expression of CD163, the scavenger receptor expressed on M2 suppressive microphages, only in the presence of cetuximab (*p* = 0.004, Fig. 5a and b). Similar changes of surface markers on CD14^+^ monocytes were also observed in the co-cultures of tumor cells and purified monocytes with cetuximab in the absence of NK cells (Fig. 5c and d), indicating M1/M2 skewing of naïve monocytes altered by cetuximab is NK-independent.

### Cetuximab down-regulates surface expression of CD16 on monocytes only in the presence of EGFR-expressing tumor cells

Activation of NK cells by cross-linking of CD16 with cetuximab-coated tumor cells resulting in loss of surface CD16 due to internalization [23, 24]. Thus, we investigated whether monocytes can be activated by cetuximab-coated tumor cells in a similar CD16-dependent manner. Surface expression of CD16 was analyzed on CD14^+^ monocytes from PBMC and purified monocytes from HNSCC patients cultured with or without PCI15B or JHU029 in the presence of 10 μg/mL cetuximab or hIgG1. CD14^+^ monocytes showed significantly decreased CD16 surface expression when cultured with cetuximab-coated tumor cells (Fig. 5e, *p* = 0.004), concomitant with upregulation of M1 and down-regulation of M2 markers (Fig. 5a-d). In contrast, PBMC or purified monocytes treated with cetuximab in the absence of EGFR-expression tumor cells did not show altered CD16 surface expression (Fig. 5f) and no difference in M1 and M2 markers were observed (data not shown). These findings indicate that cetuximab-coated tumor cells polarize naïve monocytes to M1 cells by direct crosslinking of CD16 on the monocytes by the hIgG1 Fc portion of cetuximab.

### Cetuximab down-regulates surface expression of CD16 on monocytes only in the presence of EGFR-expressing tumor cells

**Fig. 6:**
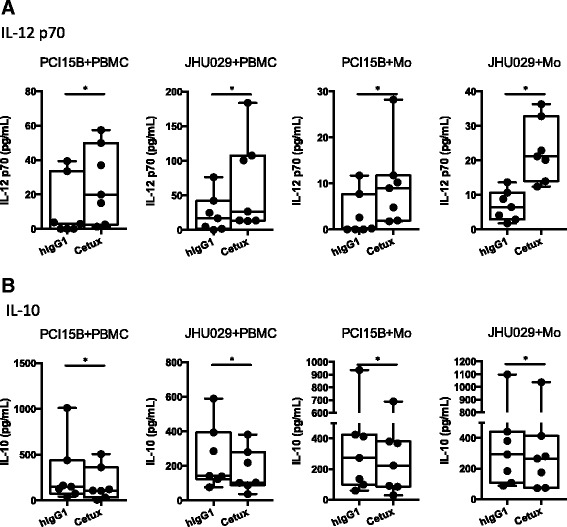
IL-12 p70 was increased and IL-10 was decreased in the supernatants of the co-cultures of tumor cells and PBMC or purified monocytes in the presence of cetuximab. Supernatants of the PBMC/monocyte + tumor co-culture described above were collected and subjected to measurement of IL-12 p70 and IL-10 by ELISA. Summary data of IL-12 p70 (**a**) and IL-10 (**b**) in the supernatants of cultures of PCI15B+ PBMC, JHU029+ PBMC, PCI15B+ purified monocytes and JHU029+ purified monocytes with 10 μg/mL hIgG1 or cetuximab is shown. The graphs present the experiments from 8 different HNSCC patients with new active diseases. Statistical significance was determined by Wilcoxon matched-pairs signed rank tests. *p < 0.05

Since production of IL-12 p70 and IL-10 are key functional mediators of M1 and M2 cells [25], respectively, we also examined IL-12 p70 and IL-10 cytokines in the supernatants of co-culture between tumor cells and PBMC or purified monocytes, incubated with or without cetuximab. PBMC or purified monocytes from HNSCC patients co-cultured with tumor cells had increased production of IL-12 p70 (Fig. 6a, *p* = 0.02) and decreased production of IL-10 (Fig. 6b, *p* = 0.02) in the presence of cetuximab. Taken together, our data suggest that cetuximab can skew naïve monocytes toward an M1-polarized phenotype when co-cultured with EGFR^+^ tumor cells, including upregulation of M1 surface markers and cytokines and reduced M2 characteristics.

## Discussion

Pander et al. previously suggested that M2 macrophages are activated in the tumor microenvironment by addition of cetuximab to bevacizumab and chemotherapy in a randomized phase III clinical trial of metastatic colorectal cancer (CRC) patients [26], which seems to contradict with our current findings. However, the actual correlation of M2 macrophages in the tumor microenvironment with cetuximab treatment was not shown. In addition, their study was a combinational therapy of cetuximab, bevacizumab and chemotherapy, instead of a single agent therapy of cetuximab as we present here. Therefore, we cannot rule out the possibility that their *in vivo* observations are a complex result of multiple therapeutic interventions, rather than cetuximab alone. Instead, in our study, we took advantage of a prospective cetuximab single agent trial (UPCI 08–013) to explore the effects of cetuximab on myeloid cells *in vivo* and demonstrated that cetuximab treatment attenuated M2 phenotypes of circulating CD11b^+^CD14^+^HLA-DR^hi^ monocytes in 08–013 responders with decreased expression of CD163 and IL-10 transcripts (Fig. 3). Pander et al. used *in vitro* differentiated M2 macrophages to co-culture with cetuximab-coated tumor cells instead of naïve monocytes as we did in this study. The upregulation of PD-L1 and IL-10 expression on M2 macrophages might be due to general activation of monocytes instead of specific skewing towards M2 by cetuximab. In our *in vitro* co-culture studies, we observed upregulation of PD-L1 but decreased production of IL-10 and increased production of IL-12 p70 in the presence of cetuximab-coated tumor cells (Figs. 5 and 6), indicating cetuximab coated on tumor cells skews naïve monocytes towards an M1 but not M2 polarization, and PD-L1 is not a necessary marker for M2 MΦ/Mo. In addition, we used a higher concentration of cetuximab in our *in vitro* studies, (10 μg/mL versus their 1 μg/mL) and studied different disease contexts (HNSCC versus CRC), which might also contribute to the different observations. Taken together, we conclude that cetuximab could ameliorate suppressive phenotypes of antigen presenting myeloid cells, skewing them away from MDSC and M2 MΦ/Mo, which is firmly supported by both *in vivo* and *in vitro* findings.

Due to small sample size (a cohort of 29 patients) and variable preliminary nature of the patients, some of our analyses only showed a trend of increase or decrease, but did not turn out to be statistically significant. Thus, we were not able to segregate the patients into different groups according to certain characteristics. Therefore, those findings might not be convincing enough to be applied to all the patients and require validation in independent cohorts. However, we still observed some consistent changes in responders or non-responders after cetuximab treatment, such as decreased granulocytic MDSC in the PBMC of responders (decreased in every responder patients after cetuximab treatment). These findings can be used as biomarkers for prognosis. Moreover, since this trial is a cetuximab single agent trial, we can infer much more clear indications about the immunologic effects of cetuximab alone on the patients, without complicating effects of chemotherapy and/or radiotherapy.

Activated NK cells are able to enhance DC maturation and mature DC can secrete IL-12 to elongate the duration of NK activation, as a reciprocal crosstalk mechanism of NK: DC crosstalk [6, 27]. Therefore, a question is raised whether cetuximab-mediated attenuation of suppressive phenotypes of myeloid cells is dependent on NK cells. Notably, the purified CD14^+^ monocytes (purity: up to 97 %) could be skewed to M1 phenotypes with increased expression of M1 surface markers and production of IL-12 p70 even in the absence of NK cells. In addition, down-regulation of CD16, but not CD32a/b, was observed on in vitro induced MDSC with FcγR: hIgG1 ligation (Fig. 4c) and monocytes co-cultured with cetuximab-coated tumor cells (Fig. 5e), indicating CD16 is internalized after interacting with the hIgG1 Fc portion of cetuximab and hIgG1 mainly interact with CD16 instead of CD32a or CD32b. In conclusion, our data suggest skewing of myeloid cells away from suppressive phenotypes by cetuximab is NK-independent. However, cetuximab-activated NK cells would be expected to enhance the M1 polarization of naïve monocytes with further enhanced up-regulation of HLA-DR and CD86 as well as amount of secreted IL-12 p70 (Figs. 5 and 6).

According to our findings, increased frequency of circulating monocytic MDSC is correlated to poor clinical response, though we also observed higher frequency of circulating monocytic MDSC in responders at baseline level (Fig. 2). We also noted that a higher frequency of Treg have been observed at baseline in cetuximab clinical responders compared to non-responders [28]. It is possible that responders have stronger initial induced anti-tumor immune responses and concomitantly more immunosuppressive cells (MDSC and Treg) at baseline (before cetuximab treatment). Besides, decreased M2 polarization of CD11b^+^CD14^+^HLA-DR^hi^ monocytes in the peripheral circulation was also correlated with better clinical outcome (Fig. 3), which is consistent with previous reports that increased abundance of MDSC and M2 MΦ/Mo correlated with poor prognosis in cancer patients [10, 29]. In conclusion, cetuximab enhances anti-tumor immune responses in HNSCC patients not only by activating NK-mediated ADCC, but also by ameliorating suppressive phenotypes of myeloid antigen presenting cells, which is associated with better outcome in treated HNSCC patients. Since circulating monocytic MDSC were more abundant in patients did not respond to cetuximab treatment, MDSC depletion or inhibition reagents should be considered to use in combination with cetuximab to improve the efficacy of cetuximab therapy.

## Conclusion

In this study, we provide a novel insight for how cetuximab modulates the immune system in HNSCC patients by ameliorating the MDSC and M2 immunosuppression, and its clinical significance, based on our *in vivo* and *in vitro* observations. First, we demonstrate increased frequency of circulating monocytic MDSC in non-responders and reduced abundance of circulating granulocytic MDSC in cetuximab-treated HNSCC patients compared to pretreatment levels. Second, CD11b^+^CD14^+^HLA-DR^hi^ monocytes in the peripheral blood of cetuximab responders showed less M2 polarization with decreased expression of CD163 and IL-10 transcripts. Third, our *in vitro* FcγR: hIgG1 ligation was able to reverse the immunosuppression of the IL-6 and GM-CSF induced MDSC, indicating the effect of cetuximab on the development of MDSC is FcγR-dependent. Fourth, cetuximab skewed naïve monocytes towards M1 phenotypes, with upregulation of surface expression of HLA-DR and CD86 and production of IL-12 p70 as well as decreased M2 characteristics (expression of surface CD163 and IL-10 cytokine), concomitant with down-regulation of surface CD16 expression. These data suggest an important role for therapeutic tumor-targeted mAb in promoting immunostimulatory phenotypes of APC in treated patients.

## Methods

### Patients and specimens

All patients were seen in the Department of Otolaryngology at the University of Pittsburgh Medical Center, and specimens from patients were obtained by informed consent under the IRB approved UPCI protocol 99–069. This study was approved by University of Pittsburgh IRB and it was a single site trial. PBMC were obtained from HNC patients receiving neoadjuvant cetuximab on a prospective phase II clinical trial (UPCI #08-013, NCT 01218048). Tumors were biopsied immediately before, and again after 4 weeks, of cetuximab therapy. Clinical response was analyzed by comparing paired CT scans pre/post cetuximab, and quantifying tumor measurement by a dedicated head and neck radiologist blinded to patient status. Anatomic tumor measurements were recorded in two dimensions and the cohort segregated into clinical “responders,” who demonstrated reduction in tumor volume, or “non-responders,” whose tumors grew during this therapy. This is a validated method to correlate clinical response, and this was the primary, pre-specified endpoint in the clinical trial (UPCI #08-013), funded and peer-reviewed by NIH R01 DE019727. Furthermore, the approach has been published [28, 30, 31]. Traditional RECIST criteria are not appropriate for short “window of opportunity” (neoadjuvant) biomarker therapeutic trials seeking to identify earlier response biomarkers associated with objective clinical response. Clinico-pathologic features of the responders and non-responders are described in Table 1.

**Table 1:** Summary of clinicopathological features of the responders and non-responders on the UPCI 08-013 trial

	No. of patients	Gender	Mean age	Tumor site	HPV status
Responders	10	Male: 6	60	OC: 8	HPV+: 1
		Female: 4		OP: 2	HPV-: 9
Non-responders	19	Male: 14	59	OC: 8	HPV+: 9
		Female: 5		OP: 9	HPV-: 10
				L: 2	

Peripheral venous blood samples were collected from a cohort of 9 stage III/IV HNSCC patients with newly diagnosed, untreated disease for in vitro studies of tumor cell and PBMC/monocyte studies.

### Collection of peripheral blood mononuclear cells (PBMC)

Venous blood from HNSCC patients was drawn into heparinized tubes and centrifuged on Ficoll-Hypaque gradients (GE Healthcare Life Sciences, Piscataway, NJ). PBMC were recovered, washed in RPMI-1640 medium (Sigma, St. Louis, MO), and either used immediately for experiments or resuspended in freezing media containing 10 % DMSO and 90 % FBS, transferred to Mr. Frosty containers (Thermo Scientific, Waltham, MA), and stored at −80 °C. Frozen PBMC were thawed in 37 °C waterbath and transferred to 15 mL conical tube. Pre-warmed RPMI-1640 medium containing 10 % FBS was added dropwise to the thawed cells. After centrifuging the cells to remove the freezing media, the cells can be used for flow cytometry or in vitro experiment.

### Antibodies and flow cytometry

The following anti-human antibodies were used for staining: CD68-FITC, CD14-PercP-Cy5.5, HLA-DR-APC, CD15-PE-Cy7, CD11b-PercP-Cy5.5, CD163-BV421, CD14-APC, HLA-DR-PE, CD33-BV421 and PD-L1-BV421 purchased from Biolegend (San Diego, CA), CD11b-PE, CD3-Alexa Fluor 700, CD19-Alexa Fluor 700, CD19-Alexa Fluor 700, CD163-Alexa Fluor 647, CD16-PE-Cy7, CD3-PE, CD19-PE and CD56-PE purchased from BD Biosciences (San Jose, CA), CD14-PE-TR and CD16 PE-TR purchased from Life Technologies (Carlsbad, CA) and CD86-FITC purchased from R&D systems (Minneapolis, MN). CD32-a-FITC Ab was purchased from Stemcell technologies, (Vancouver, Canada). CD-32-B (F-4) was purchased from Santa Cruz Biotechnology, Santa Cruz, CA, and secondary Ab anti-mouse F(ab’)2 was purchased from Thermo fisher scientific (MA, USA).

Intracellular staining of CD68 was performed as follows: PBMC were stained with surface marker antibodies, fixed with fixation/permeabilization buffer (eBioscience), washed, and stained for intracellular antigens in 1X permeabilization buffer. Cells were analyzed on an LSR Fortessa (BD) flow cytometer, and data analyzed using Flow Jo (Treestar, Ashland, OR). Dead cells were excluded based on viability dye staining (Zombie Aqua Fixable Viability Dye, Biolegend).

### Sorting of CD11b^+^CD14^+^HLA-DR^hi^ circulating monocytes from patients and quantitative real-time PCR

PBMC from HNSCC patients was obtained pre- and post-cetuximab treatment (on the UPCI 08–013 trial Table 2) were thawed and stained with CD11b-PercP-Cy5.5, CD14-APC and HLA-DR-PE antibodies. CD11b + CD14 + HLA-DRhicells were sorted using Beckman Coulter MoFlo Astrios (Brea, CA). Sorted cells were then lysed in RLT lysis buffer and subjected to RNA purification using RNeasy Mini Kit (Qiagen, Valencia, CA) and Purelink DNase Set (Invitrogen, Grand Island, NY). The concentration and purity of RNA was determined by measuring absorbance at 260 and 280 nm. RNA was used for first strand cDNA synthesis using random hexamers and MultiScribe reverse transcriptase (Applied Biosystems, Foster City, CA) according to manufacturer’s instructions. PCR probes for IL-12B (Hs01011518_m1), IL-10 (Hs00961622_m1) and GUSB (Hs99999908_m1) were purchased from Applied Biosystem for TaqMan® Gene Expression Assay. Real-time PCR cycling was performed using StepOne™ Real-Time PCR Systems (Applied Biosystems, Carlsbad, CA). GUSB was amplified as an internal control. All of the experiments were performed in triplicates. Relative expression of the target genes to endogenous control gene (GUSB) was calculated using the ΔC_T_ method: relative expression = 2^−ΔCT,^ where ΔC_T_ = C_T_ (target gene) − C_T_ (GUSB).

**Table 2:** Clinicopathological features of the 29 HNSCC patients on the UPCI 08-013 trial

Patient	Gender	Age	Tumor site	T-stage, clinical	N-stage, clinical	M-stage, clinical	T-stage, path	N-stage, path	M-stage, path	HPV status
1	Male	31	OC	T3	N0	M0	T2	N1	M0	-
2	Male	51	OC	T4	N1	M0	T4A	N0	M0	-
3	Male	48	OP	T2	N2	M0	T3	N2B	MX	+
4	Female	68	OC	T4A	N1	M0	T4A	N0	MX	+
5	Male	62	OP	T1	N1	M0	T1	N2A	MX	+
6	Male	69	OP	T2	N2	M0	T3	N2B	MX	+
7	Female	75	OC	T4	N0	M0	T4A	N0	MX	-
8	Male	65	OC	T2	N2	M0	T1	N2A	MX	-
9	Male	49	OP	T1	N2B	M0	T2	N1	MX	+
10	Female	40	OC	T2	N2B	M0	T2	N2B	MX	-
11	Female	58	OC	T3	N2B	M0	T2	N2B	MX	-
12	Female	83	OC	T4A	N0	M0	T4A	N0	MX	-
13	Male	55	L	T3	N1	M0	T3	N1	MX	-
14	Male	64	OC	T1	N1	M0	T1	N1	MX	-
15	Female	51	OP	T4	N0	M0	T2	N0	MX	-
16	Male	59	OP	T2	N2B	M0	T2	N2A	MX	+
17	Male	64	OP	T2	N1	M0	T2	N2B	MX	+
18	Male	62	OC	T2	N0	M0	TX	N0	MX	-
19	Male	54	OC	T2	N2C	M0	T2	N2C	MX	-
20	Male	62	OC	T4	N1	M0	T2	N1	MX	-
21	Male	93	OC	T4A	N0	M0	TX	NX	MX	-
22	Male	58	OP	T2	N1	M0	T3	N1	MX	-
23	Female	47	OC	T4A	N1	M0	T4A	N1	MX	-
24	Male	59	OC	T4A	N0	M0	T4A	N0	MX	-
25	Male	64	OP	T2	N1	M0	T1	N1	MX	+
26	Male	43	OP	T2	N2B	M0	T2	N2B	MX	+
27	Female	74	L	T4	N1	M0	T3	N2C	MX	-
28	Female	57	OC	T4A	N1	M0	T2	N1	MX	-
29	Male	57	OP	T3	N2B	M0	T1	N0	MX	+

*Abbreviations: OC* oral cavity, *OP* oropharynx, *L* larynx

### hIgG1 ligation assay

10 μg/mL human IgG1 (Southern Biotech, Birmingham, AL) dissolved in 1X ELISA coating buffer (Biolegend, San Diego, CA) was added to T-75 flasks. Then the flasks were incubated for 2 h at 37 °C or overnight at 4 °C. Coated flasks were washed with sterile PBS 3 times before use.

### In vitro monocyte induction of MDSC and MDSC suppression assay

PBMC isolated from healthy donors (*n* = 3) were plated in the hIgG1-coated flasks or uncoated flasks at 5X10^5^ cells/mL in complete medium for 7 days, supplied with or without 10 ng/mL IL-6 and GM-CSF (R&D systems, Minneapolis, MN). Medium and cytokines were refreshed every 2–3 days. After 7 days, all cells were harvested from the cultures. Adherent cells were removed using trypsin. Some of the harvested cells were subjected to analysis of surface markers by flow cytometry. The other cells were stained with CD33-BV421, CD3-PE, CD19-PE and CD56-PE and CD3^−^CD19^−^CD56^−^CD33^+^ cells from each culture were isolated using FACS sorting (Beckman Coulter MoFlo Astrios, Brea, CA).

Fresh T cells were isolated from PBMC of autologous donors by Human T cell Enrichmen Kit (Stemcell Technologies, Vancouver, Canada) and were CFSE-labeled (2 μM, Life Technologies, Carlsbad, CA) and seeded in 96-well plates at 1X10^5^ cells per well. CD33^+^ cells isolated from the cultures indicated above were added to these wells at a 1:2 ratio of CD33^+^ cells: T cells. T cell stimulation was provided by anti-CD3/CD28 stimulation beads (Invitrogen, Carlsbad, CA) at a 1:1 ratio of T cells: beads. Suppression assay wells were analyzed by flow cytometry for T cell proliferation after 3 days.

### Tumor cell: PBMC/purified monocyte co-culture studies

PBMC were isolated from 9 HNSCC patients with newly diagnosed diseases. CD14^+^ monocytes were purified from PBMC using EasySep human CD14 positive selection kit (Stemcell Technologies, Vancouver, Canada). PCI15B or JHU029 were seeded in 96-well plates at 1X10^4^ cells per well. PBMC were added to these cells at a ratio of 1:10 (tumor cells: PBMC) and purified monocytes were added at a ratio of 1:5 (tumor cells: monocytes) with 10 μg/mL cetuximab or hIgG1 (Southern Biotech, Birmingham, AL). Cells were harvested for flow cytometry analysis of surface markers and supernatants were collected and stored in −80 °C after cultured for 3 days.

### ELISA

IL-12 p70 and IL-10 in the supernatants of the co-cultures indicated above were tested using Human IL-12 p70 ELISA Kits and Human IL-10 ELISA Kits (Thermo Scientific, Rockford, IL), per manufacturer’s instructions.

### Statistical analysis

Averages were calculated as means. Two-group comparison was performed using paired Wilcoxon signed rank text in GraphPad Prism (GraphPad, La Jolla, CA). Experimental data with more than 2 comparison groups were analyzed using one-way ANOVA with post hoc pairwise comparisons using Tukey’s multiple comparison test. *P*-values < 0.05 were considered to be significant. *p < 0.05, **p < 0.01.

## Additional file

Additional file 1: Figure S1. Gating strategies of monocytic MDSC and granulocytic MDSC in the peripheral blood. Gating strategies of flow cytometry analysis of monocytic MDSC (CD11b^+^CD14^+^HLA-DR^lo/-^) and granulocytic MDSC (Lin^−^CD11b^+^CD15^+^) from HNSCC patients were plotted in A and B, respectively. **Figure S2.** Similar baseline expression of CD16 on circulating monocytic MDSC in 08–013 responsers and non-responders. Summary data of frequency of CD16+ cells and MFI of CD16 in CD14^+^HLA-DR^lo/-^ cells in responders (*n* = 10) and non-responders (*n* = 19) of UPCI 08–013 trial pre-cetuximab treatment. Statistical significance was determined by Mann Whitney test. (PPTX 553 kb)

## Abbreviation

MDSC: Myeloid-derived suppressor cells
mAb: Monoclonal antibodies
EGFR: Epidermal growth factor receptor
FcγR: Fc-gamma receptors
HNSCC: Head and neck squamous cell carcinoma (HNSCC)
MLR: Mixed leukocyte reaction
ADCC: Antibody dependent cell-mediated cytotoxicity
APC: Antigen presenting cells
ROS: Reactive oxygen species
ITAM: Immunoreceptor tyrosine-based activation motif
PBMC: Peripheral blood mononuclear cells
hIgG1: Human IgG1
