# Supplementary figures and images for: Cetuximab ameliorates suppressive phenotypes of myeloid antigen presenting cells in head and neck cancer patients
Source: J Immunother Cancer. 2015 Nov 17;3:54. doi: 10.1186/s40425-015-0097-6 (PMC4647471; doi:10.1186/s40425-015-0097-6)

## Slide 1
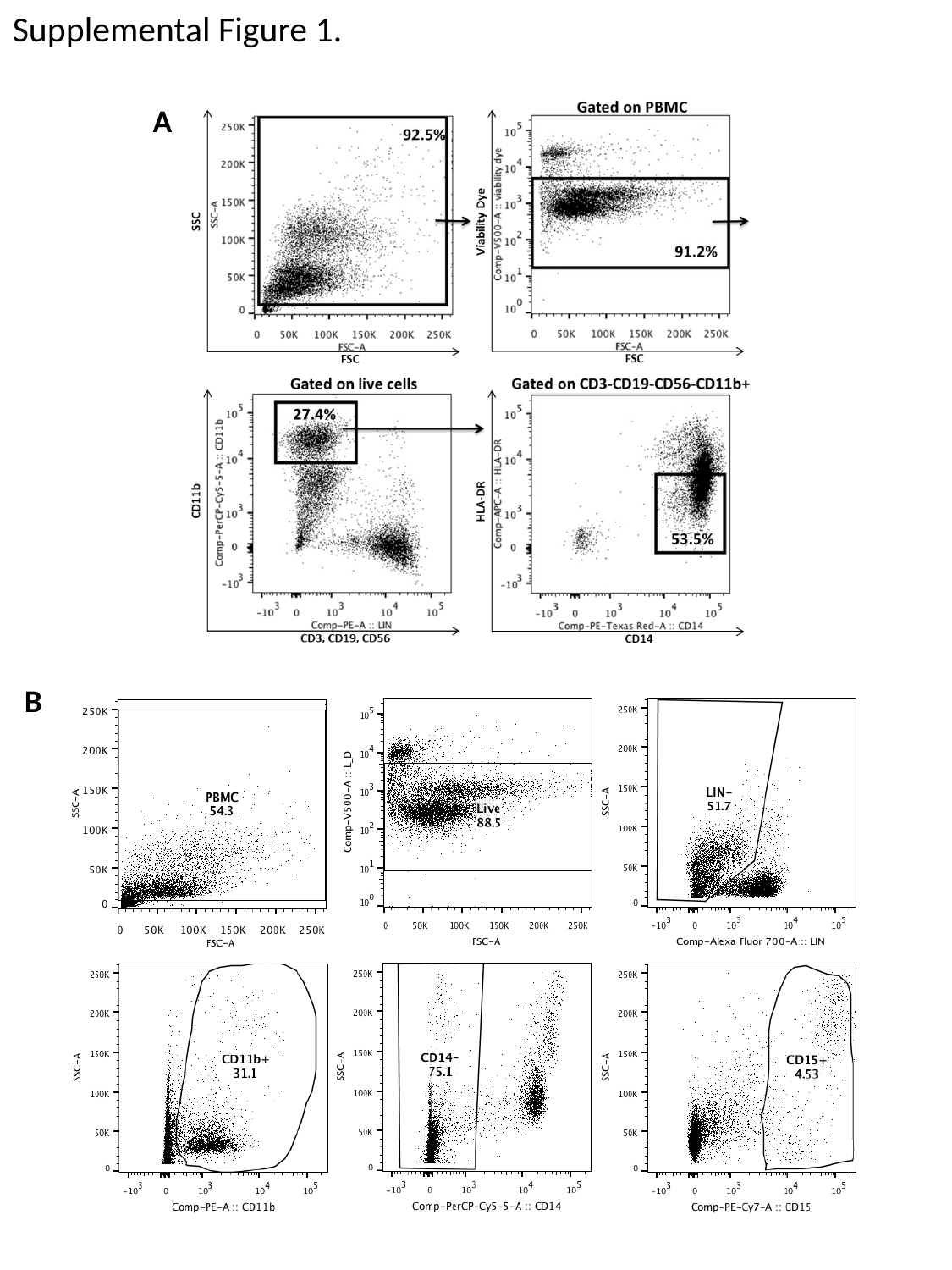

Supplemental Figure 1.
A
B

## Slide 2
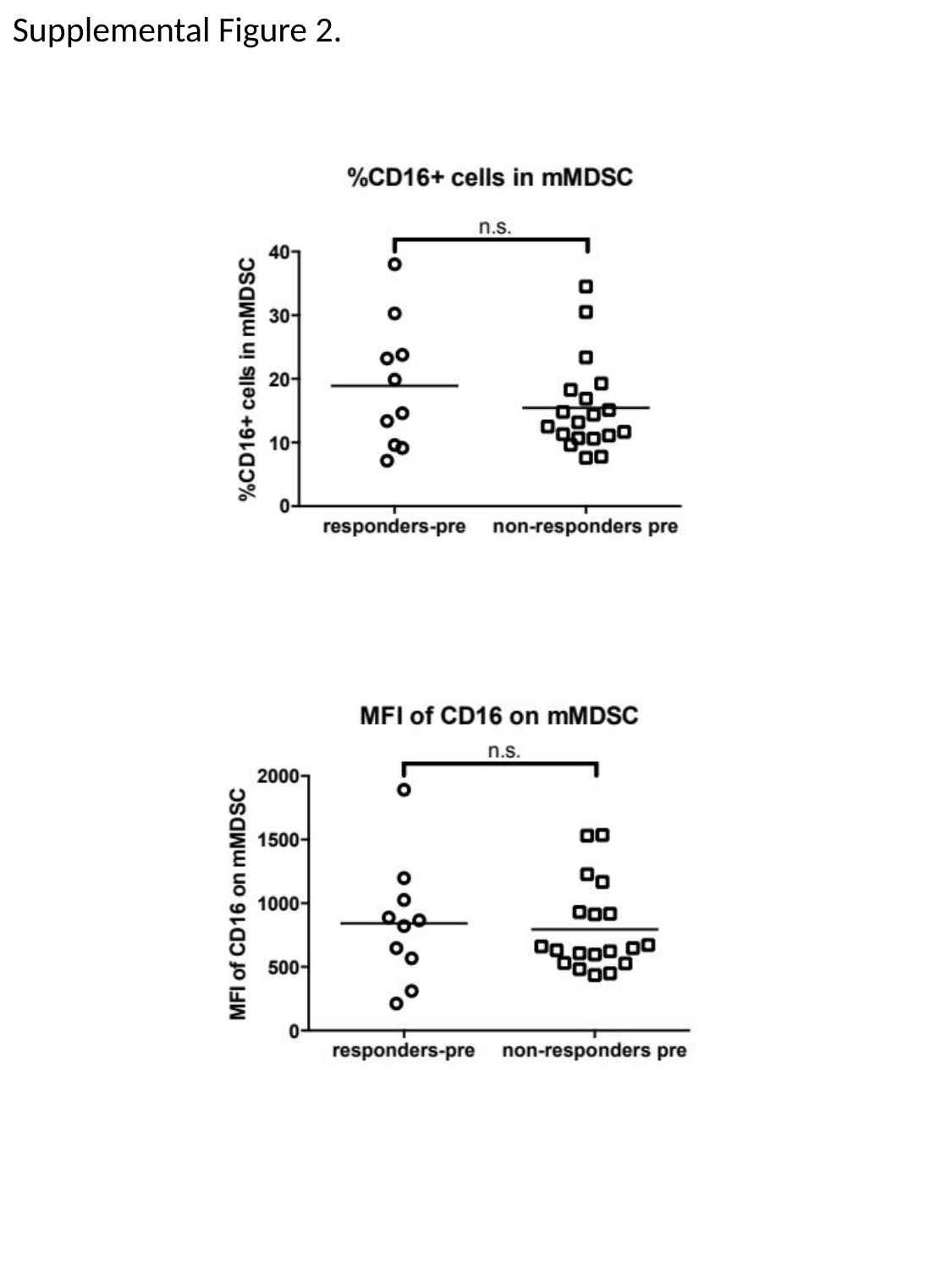

Supplemental Figure 2.

Supplement: Additional file 1: Figure S1. — Gating strategies of monocytic MDSC and granulocytic MDSC in the peripheral blood. Gating strategies of flow cytometry analysis of monocytic MDSC (CD11b+CD14+HLA-DRlo/-) and granulocytic MDSC (Lin−CD11b+CD15+) from HNSCC patients were plotted in A and B, respectively. Figure S2. Similar baseline expression of CD16 on circulating monocytic MDSC in 08–013 responsers and non-responders. Summary data of frequency of CD16+ cells and MFI of CD16 in CD14+HLA-DRlo/- cells in responders (n = 10) and non-responders (n = 19) of UPCI 08–013 trial pre-cetuximab treatment. Statistical significance was determined by Mann Whitney test. (PPTX 553 kb) [file 40425_2015_97_MOESM1_ESM.pptx]
